# Acute effect of dark chocolate intake before high-intensity resistance exercise on arterial stiffness in healthy young men

**DOI:** 10.1016/j.jesf.2025.08.005

**Published:** 2025-08-25

**Authors:** Urara Hata, Yuto Hashimoto, Midori Natsume, Takanobu Okamoto

**Affiliations:** a Graduate School of Health and Sport Science, Nippon Sport Science University, Tokyo, Japan; b Research Institute for Sport Science, Nippon Sport Science University, Tokyo, Japan; c Food Microbiology and Function Research Laboratories, Meiji Co., Ltd., Tokyo, Japan; d Faculty of Sport Science, Nippon Sport Science University, Tokyo, Japan

**Keywords:** Dark chocolate, Cocoa polyphenol, Pulse wave velocity, Arterial compliance, Beta stiffness index, Resistance exercise

## Abstract

**Aims:**

This study investigated the effect of dark chocolate (DC) intake before resistance exercise (RE) on arterial stiffness.

**Methods:**

Twelve healthy adult males (age, 23.0 ± 1.0 years) performed DC or white chocolate (WC) intake trial on separate days in a randomized crossover trials. Participants consumed 50g of DC containing 1285 mg cocoa polyphenols or an isocaloric amount of WC 50g without polyphenols. All participants performed 5 sets of 5 repetitions using 80 % of the 1 repetition maximum (1RM) bench press and 5 sets of 10 repetitions using 70 % of the 1RM biceps curl. Brachial–ankle pulse wave velocity (baPWV), blood pressure, and heart rate were measured before chocolate intake (baseline); and before (at 60 min after chocolate intake), immediately after, and at 30 and 60 min after completing the RE.

**Results:**

In both trials, there was a significant increase in baPWV immediately after the RE compared with baseline (baseline: DC 1103 ± 94 cm/s, WC 1108 ± 167 cm/s; immediately after RE: DC 1300 ± 187 cm/s, WC 1325 ± 178 cm/s; P < 0.05). In the DC intake trial, baPWV decreased to 1210 ± 180 cm/s at 30 min and 1155 ± 134 cm/s at 60 min after RE, compared to immediately after RE (P < 0.05). In contrast, in the WC intake trial baPWV was decreased to 1222 ± 176 cm/s at 60 min after RE compared to immediately after RE (P < 0.05).

**Conclusion:**

These results suggest that in comparison with WC intake, DC intake before RE might quickly reduce post-exercise increased arterial stiffness. **Clinical Trials Registry Number**: UMIN000052616.

## Abbreviations:

ANOVA: analysis of variance
baPWV: brachial-ankle pulse wave velocity
BMI: body mass index
BP: blood pressure
DC: dark chocolate
ET-1: endothelin-1
FMD: flow mediated dilation
NO: nitric oxide
NSCA: national strength and conditioning association
RE: resistance exercise
SD: standard deviation
WC: white chocolate
1RM: 1 repetition maximum

## 1 Introduction

Increases in arterial stiffness is an independent risk factor and has been reported to predict not only the risk of cardiovascular mortality, but also death, contributing to all causes of death in hypertensive patients.^1^^,^^2^ Arterial stiffness increases with age, lifestyle-related diseases, and smoking.^3^^,^^4^ The challenge is to control the increase in arterial stiffness because it increases blood pressure, interferes with the efficient circulation of blood, and increases the risk of developing cardiovascular disease.^1^

Moderate-to-high-intensity resistance exercise (RE) (approximately 65–85 % of 1 Repetition Maximum: 1RM) is an effective intervention for the prevention of sarcopenia, osteoporosis, and lifestyle-related diseases, and is therefore recommended for implementation.^5^ However, many previous studies have reported that moderate-to-high intensity RE increases arterial stiffness.^6^, ^7^, ^8^, ^9^ Pulse wave velocity (PWV), an index of arterial stiffness, has been reported to increase after acute and habitual RE. A study of young men who performed high-intensity RE reported decreases and increases in arterial compliance and beta stiffness index, respectively, which was maintained until 30–60 min after the RE.^6^ As it has been suggested that performing moderate-to-high-intensity RE places stress on the cardiovascular system, it is necessary to identify ways to reduce the increase in arterial stiffness after RE.

Dark chocolate (DC) has a very high cocoa-polyphenol content^10^ and is an efficient source of polyphenols. Both acute and habitual intake of DC has been shown to reduce blood pressure, decrease arterial stiffness, and improve vascular endothelial function.^11^, ^12^, ^13^, ^14^ Many studies have shown that acute intake of flavanol-rich DC improves vascular endothelial function,^15^^,^^16^ which has been compared to flavanol-free WC. In fact, it has been reported that DC ingestion improves vascular endothelial function after 2 h.^12^ Numerous studies have shown that acute ingestion of DC improves vascular function and has an immediate effect.^12^^,^^14^ However, whether DC ingestion can inhibit arterial stiffness increased by high-intensity RE (defined as ≧70 % 1 RM) has not been examined.

Therefore, the aim of this study was to investigate the effect of intake of DC rich in cacao polyphenols and that of white chocolate (WC) without cocoa polyphenols on arterial stiffness after high-intensity RE in healthy young men. We hypothesized that DC consumption before RE would have greater effect in reducing increases in arterial stiffness after high-intensity RE compared to WC consumption.

## 2 Methods

### 2.1 Participants

A priori power analysis was performed using G∗Power (version 3.1.4; Heinrich-Heine University, Düsseldorf, Germany) to determine the minimum required sample size. Assuming an effect size of 0.25 (medium), a significance level of α = 0.05, power (1–β) = 0.80, and repeated measures ANOVA with five time points (baseline, before resistance exercise, immediately after, 30 min after, and 60 min after exercise) across two conditions (dark chocolate and white chocolate), the analysis indicated that at least 11 participants were required. Therefore, a total of 14 participants were recruited to account for potential dropouts. Two participants were excluded due to withdrawal due to ill health and injury, leaving 12 participants for the final analysis. The mean ± standard deviation values for the age, height, body mass, body fat percentage, and body mass index (BMI) were 23.1 ± 1.0 years, 172.6 ± 5.6 cm, 67.5 ± 6.9 kg, 14.5 ± 3.8 %, and 22.6 ± 1.4 kg/m^2^, respectively. All participants had normal blood pressure (BP) (<120/80 mmHg) and no obvious signs, symptoms, or history of chronic disease. Body composition was assessed using a bioelectrical impedance analyzer (InBody 770, Biospace Co., Seoul, Korea). Participants had no resistance or aerobic exercise habits, which was determined using the international Physical Activity Questionnaire (IPAQ). They had not smoked or taken any medications for at least 1 year. Measurements for this study were conducted from April to September 2022. Female participants were not included in this study to rule out the influence of the menstrual cycle on changes in arterial stiffness after intense exercise.

This study was conducted in accordance with the Declaration of Helsinki and approved by the Ethics Committee of the Nippon Sport Science University (021-H244). This study is registered in the Japanese Clinical Trials Registry (UMIN000052616). All participants were informed in detail about the purpose of the study, the experimental procedures, and all potential risks. Written informed consent was obtained from each participant prior to the start of the experiment.

### 2.2 Body composition

Height was measured to the nearest 0.1 cm using a stadiometer (YG-200; Yagami, Tokyo, Japan). Weight and body fat were measured using a body composition analyzer (Inbody 770, Biospace Co., Seoul, Korea) by the impedance method.

### 2.3 Study design

Participants were instructed to refrain from any exercise, therapy (massage, stretching, etc.), or medication during the experimental period. All participants were asked to limit their intake of polyphenols one week before being tested to minimize any of their potential effects. Apart from this restriction, they were instructed to maintain their usual diet throughout the study period, and no other special dietary restrictions were imposed. In addition, all participants were instructed to refrain from engaging in strenuous exercise, binge eating, caffeine intake, and alcohol consumption on the day before measurements were obtained. To eliminate any potential effects of food intake, they were instructed to fast from 12 h before measurement. Each trial was conducted in a randomized crossover fashion. The order of trials was determined by an independent third party using a random sequence generated in Microsoft Excel. Trials were spaced at least one week apart.

**Fig. 1:**
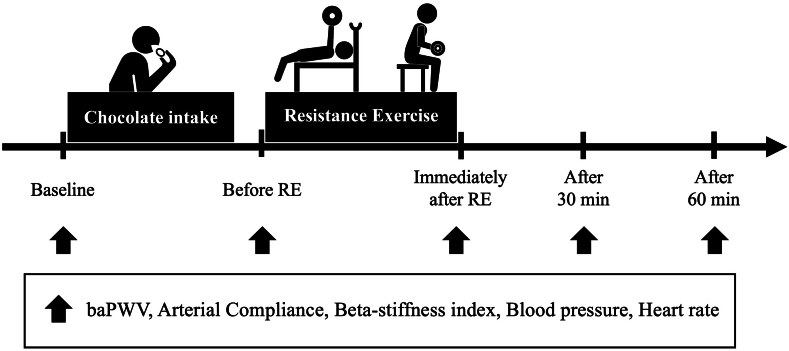
Study design.

All measurements were performed in the morning (9:00–13:00). The measurement room was set at 24–26 °C. Each participant rested in the supine position for a minimum of 10 min after arrival at the laboratory. Brachial-ankle PWV (baPWV), arterial compliance, and beta-stiffness index were then measured. After the baseline measurements, participants ingested either DC or WC 60 min before RE. Chocolate was ingested 60 min before RE to align the timing of the post-exercise arterial stiffness measurements, which began 30 min after exercise, with the expected peak in plasma polyphenol concentration occurring approximately 120 min after ingestion 6, 17, 18. Participants were asked to remain seated and at rest until the next measurement. They were placed in the supine position 10 min before the measurement. Arterial compliance, baPWV, and beta-stiffness index were measured once more immediately prior to the participants performing two REs: bench press and arm curl. These three measurements were then repeated immediately after RE, and at 30 and 60 min after RE (Fig. 1).

### 2.4 Chocolate

Participants were randomly assigned to intake of 50 g of either DC or WC on separate days in a randomized crossover trial. The DC (Meiji Co., Ltd. Tokyo, Japan) was enriched in cocoa polyphenols (1285 mg), whereas the WC (Meiji Co., Ltd.) contained no cocoa polyphenols. The dose of cocoa polyphenols (1285 mg) was selected because it represents the lowest amount reported in previous studies to elicit measurable improvements in vascular function, while still being within a practically consumable range.^13^^,^^19^ The nutrition facts of DC and WC was almost identical. The amount of cacao polyphenol was determined by the Folin Ciocalteu method using epicatechin as the standard, which is a modification of Singleton et al.’s method.^20^ Cacao procyanidins were determined as (+)-catechin, (−)-epicatechin, procyanidin B2, procyanidin B5, procyanidin C1, (+)-catechin, (−)-epicatechin, procyanidin B5, procyanidin C1, (−)-epicatechin as standard, based on Natsume et al.^21^ Procyanidin B2, procyanidin B5, procyanidin C1, and cinnamtannin A2 were quantified as (−)-epicatechin equivalents, and the total was determined as the sum. The intervention was not blinded to either the participants or the investigators bue to differences in taste and appearance between DC and WC. The analytical results and nutrition facts are summarized in Table 1.

**Table 1:** Composition of the dark and white chocolate used in the study.

Nutrients in intake(50g)	Dark Chocolate	White Chocolate
Calories (kcal)	284.6	296.4
Protein (g)	5.4	4.2
Fat(g)	20.6	20.0
Carbohydrate (g)	22.6	24.8
Cocoa polyphenol (mg)	1285	–
-Procyanidins 6 types(mg)	155	–

Procyanidins total of (+)-catechin, (−)-epicatechin, procyanidin B2, procyanidin B5, Procyanidin C1, Cinnamtanin A2.

### 2.5 Resistance exercise

All participants performed REs consisting of bench presses and biceps curls, which were effective in acutely increasing arterial stiffness.^22^^,^^23^

1RM measurements to determine the arm curl and bench press loads for all participants were obtained according to the National Strength and Conditioning Association (NSCA) instruction manual “1RM Test Protocol”.^24^ Participants warmed up with a weight with which they could comfortably perform five repetitions, rested for 1 min, and then warmed up with a weight set to three repetitions. After resting for 2 min, the weight was set to that which could be repeated twice with a near-maximum load. Three warm-up sets were performed. After resting for 4 min, the 1RM measurement was started by increasing by 4–9 kg the load on the weight with two repetitions at near maximal load. The 1RM was measured within 5 sets, and the load was increased by 2–4 kg if the subject was able to raise the bar, and decreased by 2–4 kg if the subject was unable to raise the bar. Rest between sets was set at 4 min.

All participants performed REs consisting of bench presses and biceps curls, which were effective in acutely increasing arterial stiffness. The weight for each exercise was calculated based on 1RM values. For the bench press, warm-up was performed at 1RM 50 % for 10 repetitions, followed by 5 sets of 5 repetitions at 1RM 80 %. For the arm curls, warm-up was 1RM 50 % 10 times, followed by 5 sets of 1RM 10 times. Rest periods between warm-ups, sets, and events were 120 s. Participants repeated the elevation exercise at a nearly constant speed and frequency using a metronome (2 s of elevation, 1 s of rest, and 2 s of descent). This RE protocol was selected based on previous studies that demonstrated its efficacy in inducing increases in arterial stiffness.^22^^,^^23^ To account for diurnal variation, all participants' REs were performed at the same time. A trained examiner verbally encouraged the participants and ensured that they used proper form to avoid holding their breath during the RE.

### 2.6 Pulse wave velocity (PWV)

Although the resistance exercise in this study targeted the upper limbs, we selected baPWV as the primary index of arterial stiffness because baPWV reflects both central and peripheral arterial sites and is widely used due to its practicality, reproducibility, and clinical significance. An increases in baPWV following RE have been reported previously.^23^ Since cocoa flavanols act primarily on peripheral blood vessels,^16^ baPWV was considered suitable for evaluating systemic vascular responses. Measurements were taken in a quiet measurement room set at 24–26 °C. baPWV was performed using a blood pressure pulse wave testing device (from PWV/ABI: Fukuda-Colin, Co., Ltd. Tokyo, Japan) that can simultaneously record PWV, BP, ECG and heart sounds. All participants rested in the supine position for at least 10 min before baPWV was measured. Electrocardiogram electrodes were placed on both wrists, and a phonocardiograph was placed on the left side of the sternum to detect the heartbeat. A cuff was wrapped around both the upper arm and ankle were connected to a pulse wave sensor measuring volume pulse waveform and an oscillometric pressure sensor measuring BP to measure volume pulse morphology and BP, respectively.

The distance from the aortic root to the measuring point on the upper arm (Lb) was calculated as follows: Lb = 0.2195 × participant height (cm) − 2.0734. The distance from the aortic root to the ankle (La) was calculated as follows: La = 0.8129 × participant height (cm) + 12.328. The time interval between the wave fronts of the brachial and ankle waveforms was defined as the interval between the brachium (cubital fossa) and the ankle (ΔTba). Finally, baPWV was calculated as baPWV = (La − Lb)/Tba.

### 2.7 BP and heart rate

BP and heart rate were measured simultaneously with baPWV using a BP pulse wave device (from PWV/ABI: Fukuda-Colin Co., Ltd.) from sphygmomanometer cuffs attached to both upper arms and electrodes attached to both wrists. Carotid artery BP was measured using a baroreflex tonometer fixed to the left common carotid artery by a neck collar.

### 2.8 Arterial compliance and beta-stiffness index

Measurements were performed using an ultrasound device (Vivid T8: GE Healthcare Japan, Inc., Tokyo, Japan). Longitudinal cross-sectional images of the common carotid artery 1–2 cm from the carotid sinus on the side of the aortic arch were taken in B mode using an L6-12-RS probe (GE Healthcare, Japan Inc.). The right common carotid artery was measured using an ultrasound system and applanation tonometry. The images were used to clearly record the boundary plane of the arterial vessel wall. Image analysis was measured using an ultrasound device. Systolic and diastolic vessel diameters were manually measured from longitudinal images of the cephalic portion of the common carotid artery, acquired 1–2 cm distal to the carotid bulb. Systolic and diastolic vessel diameters were measured at three locations per image from each of the 10 heartbeat images and averaged. All image analysis were performed by the same investigator.

The beta-stiffness index was calculated by dividing the systolic and diastolic vessel diameters by the systolic and diastolic blood pressures at the time of measurement. All image analyses were performed by the same examiner. Arterial compliance and beta-stiffness index were calculated as follows:

$$\text{Arterial}\;\text{compliance}=\left[\left(\text{Ds}-\text{Dd}\right)/\text{Dd}\right]/\left[2\left(\text{Ps}-\text{Pd}\right)\right]\times \pi \times {\left(\text{Dd}\right)}^{2}\text{,}$$

Beta−stiffnessindex=ln(Ps/Pd)/[(Ds−Dd)/Dd],

where Ds is maximum systolic vessel diameter, Dd is maximum diastolic vessel diameter, Ps is systolic blood pressure, Pd is diastolic blood pressure, and ln is natural logarithm ^7^.

### 2.9 Statistical analysis

All data are presented as mean ± standard deviation (SD). Analysis was performed using statistical software (IBM SPSS Statistics Ver. 27.0, IBM Co., Ltd., Tokyo, Japan). Normality tests were evaluated using the Shapiro–Wilk test. Corresponding two-way analysis of variance (ANOVA) (trial × time) was used to compare each item, and multiple comparisons using the Bonferroni method were performed as a subtest when an interaction was observed. Statistical significance was set at P < 0.05.

## 3 Results

### 3.1 baPWV

**Fig. 2:**
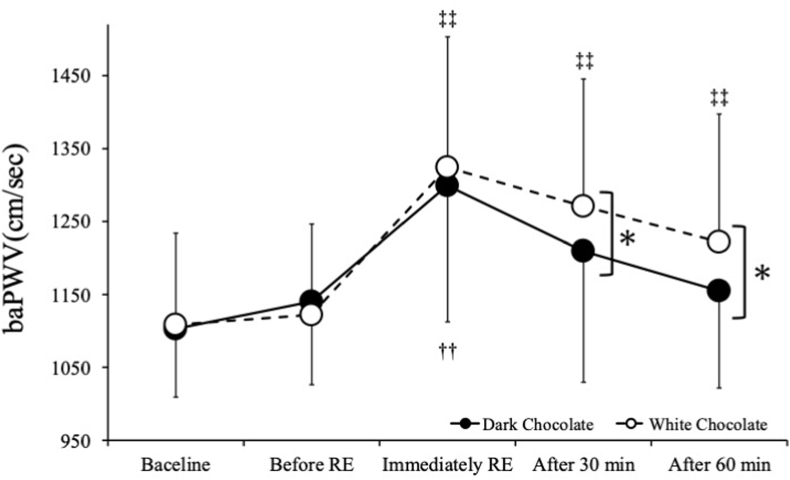
baPWV values before and after resistance exercise. Values are expressed as mean ± standard deviation. baPWV: brachial-ankle pulse wave velocity. RE: resistance exercise. ∗p < 0.05 vs. white chocolate, ††p < 0.01 vs. dark chocolate baseline, ‡‡p < 0.01 vs. white chocolate baseline.

Fig. 2 shows baPWV values before and after RE. ANOVA showed an interaction between the DC and WC intake trials (p < 0.01). At 30 and 60 min after RE, baPWV was significantly lower in the DC intake trial than the WC intake trial (p < 0.01). In the DC intake trial, baPWV was significantly lower at 30 and 60 min after RE compared to immediately after RE (p < 0.01). In the WC intake trial, baPWV was lower at 60 min after RE compared to immediately after RE (p < 0.05), but were higher immediately after RE, and at 30 and 60 min after RE compared to before RE (p < 0.05).

### 3.2 Arterial compliance

**Fig. 3:**
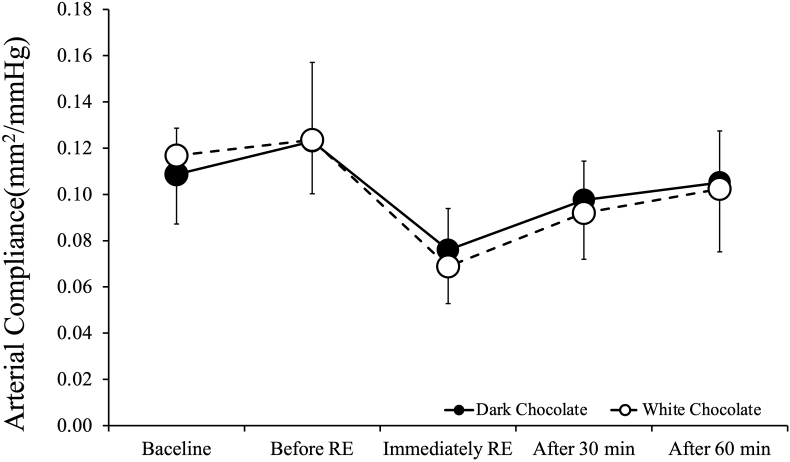
Arterial compliance values before and after resistance exercise. Values are expressed as mean ± standard deviation.

Fig. 3 shows arterial compliance values before and after RE. ANOVA showed no interaction between DC intake trials and WC intake trials in terms of arterial compliance (p = 0.28). There was no significant main effect for trial (p = 0.618), but a significant main effect for time (p < 0.001). In both trials, arterial compliance was lower than baseline immediately after RE (p < 0.001), at 30 min (p < 0.01), and at 60 min after RE (p < 0.05).

### 3.3 Beta-stiffness index

**Fig. 4:**
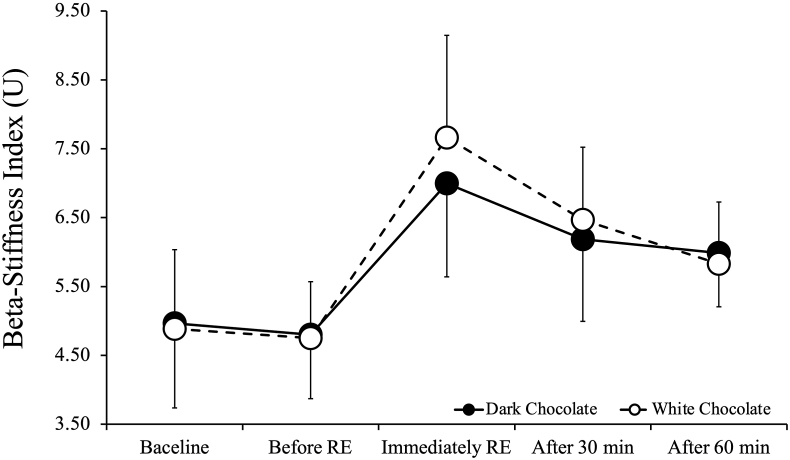
Beta-stiffness index values before and after resistance exercise. Values are expressed as mean ± standard deviation.

Fig. 4 shows beta-stiffness index values before and after RE. ANOVA showed no interaction between DC consumption trials and WC intake trials in terms of in beta-stiffness index (p = 0.19). There was no significant main effect for trial (p = 0.429), but a significant main effect for time (p < 0.001). In both trials, beta-stiffness index was higher than baseline immediately after RE (p < 0.001), at 30 min (p < 0.01), and at 60 min after RE (p < 0.05).

### 3.4 BP and heart rate

Table 2 shows changes in BP and heart rate before and after RE. ANOVA showed an interaction between the DC intake trial and WC intake trial for systolic blood pressure (p < 0.05). However, post-hoc analysis revealed no significant differences between groups at any time point. Within the s DC intake trial, values were significantly lower 60 min after RE compared to immediately after RE (p < 0.05). ANOVA showed no interaction between DC intake trial and WC intake trials in terms of MAP, diastolic blood pressure, pulse pressure, carotid SBP or heart rate (p = 0.144, p = 0.072, p = 0.841, p = 0.199, p = 0.17, respectively). In pulse pressure significant main effect for time (p < 0.001). In both trials, pulse pressure was higher than baseline immediately after RE(p < 0.001), at 30 min (p < 0.01), and at 60 min after RE (p < 0.05).

**Table 2:** Changes in blood pressure and heart rate before and after resistance exercise.

							*p* value			
Variable	Condition	Baseline	Before RE	Immediately RE	After 30 min	After 60 min	Trial	Time	Interaction	η^2^
Brachial SBP (mmHg)	DC	119.4 ± 8.6	120.9 ± 6.9	123.1 ± 5.5	121.1 ± 6.3	117.3 ± 4.9^§^	0.203	0.165	**0.017**	0.084
	WC	116.8 ± 8.6	117.3 ± 5.3	119.4 ± 7.2	118.1 ± 8.3	120.8 ± 8.1				
Brachial MAP (mmHg)	DC	85.6 ± 7.0	86.0 ± 7.4	93.4 ± 6.6	88.3 ± 6.0	85.4 ± 4.5	0.274	**0.001**	0.144	0.050
	WC	86.3 ± 6.1	83.6 ± 6.2	88.9 ± 6.7	86.4 ± 7.1	86.6 ± 7.5				
Brachial DBP (mmHg)	DC	67.0 ± 7.3	66.4 ± 5.7	64.0 ± 7.5	62.7 ± 5.3	61.6 ± 4.2	0.886	**0.007**	0.072	0.059
	WC	67.1 ± 5.1	64.9 ± 4.7	61.4 ± 4.6	61.8 ± 6.9	65.8 ± 6.4				
Brachial PP (mmHg)	DC	52.4 ± 2.9	54.5 ± 3.9	59.1 ± 7.9	58.4 ± 4.5	55.6 ± 2.4	**0.022**	**0.001**	0.841	0.007
	WC	49.6 ± 4.8	52.4 ± 4.7	58.0 ± 6.6	56.3 ± 4.9	55.0 ± 5.5				
Carotid SBP (mmHg)	DC	128.9 ± 12.7	126.5 ± 8.8	147.3 ± 10.4	134.0 ± 11.0	128.2 ± 7.8	0.220	**0.001**	0.199	0.022
	WC	125.4 ± 13.8	123.1 ± 8.8	142.7 ± 10.4	128.0 ± 10.4	131.3 ± 9.8				
Heart rate (beats/min)	DC	55.3 ± 3.7	56.2 ± 3.4	77.2 ± 6.5	66.6 ± 6.2	64.2 ± 7.8	0.084	**0.001**	0.178	0.010
	WC	54.3 ± 5.0	55.8 ± 4.7	72.0 ± 8.1	64.3 ± 5.0	60.8 ± 6.3				

Values are expressed as mean ± standard deviation. SBP: systolic blood pressure, MAP: mean arterial pressure, DBP: diastolic blood pressure, PP: pulse pressure. §p < 0.05 vs. dark chocolate Immediately after RE.

## 4 Discussion

The present study compared the effect of DC and WC intake before RE on arterial stiffness. The key finding was that increased baPWV at 30 and 60 min after RE was lower in the DC consumption trial compared to the WC consumption trial. These results suggest that DC intake before RE may have attenuated the increase in arterial stiffness caused by RE, compared to WC, although no control condition without chocolate consumption was included.

A previous study of transient high-intensity RE of the upper extremities reported an increase in baPWV of approximately 14 % immediately after RE compared to pre-exercise.^25^ In addition, a study of RE similar to the present study showed significant increases (of 10 % and 7 % at 30 and 60 min, respectively, after RE) compared to before RE.^23^ In the present evaluation, baPWV increased by 17 % and 20 % immediately after RE compared to before RE in the DC consumption and WC intake trials, respectively, consistent with the findings of Li et al.^25^ The WC intake trial also showed an increase in baPWV immediately after RE compared to before RE. In the WC intake trial, baPWV was significantly higher after 30 and 60 min of RE than before exercise by 14 % and 10 %, respectively, consistent with the findings of Okamoto et al.^23^ In contrast, in the DC intake trial, there was no significant increase in baPWV compared to baseline and before RE. baPWV increased by 9 % at 30 min and 4 % at 60 min after RE. However, compared to immediately after RE, there was a significant decrease of 10 % at 30 min and 10 % at 60 min. In the DC intake trial, baPWV was significantly higher immediately after RE compared to before RE, and decreased significantly after 30 and 60 min of RE. These results suggest that consumption of DC prior to RE effectively reduced the arterial stiffness that had been increased by performing RE, in comparison with WC.

In this study, the only significant trial-to-trial difference after RE was baPWV. No significant trial-to-trial difference was found in arterial compliance or beta-stiffness index. The present results suggest that DC intake before RE caused a reduction in systemic arterial stiffness but did not affect central arterial stiffness. Although brachial pulse pressure (PP) appeared slightly higher in the DC condition compared to the WC condition, no significant interaction effect was observed between trials (p = 0.841). This suggests that the apparent difference in PP between conditions is unlikely to reflect a physiologically meaningful response. While cocoa flavanols have been reported to acutely influence vascular function, such effects were not evident in our data. In a previous study, arterial compliance and beta-stiffness index were reported to be significantly decreased and significantly increased immediately after RE, with lower or higher values until 30 and 60 min, respectively, after RE.^6^ We found that arterial compliance was lower immediately and at 30 and 60 min after RE compared to before RE in both intake trials, and beta-stiffness index was higher, consistent with the findings of DeVan et al.^6^ Thus, our results suggest that DC intake before RE may not affect central arterial stiffness.

The effects of DC intake on arterial stiffness have been reviewed by Ludovici et al.^13^ In addition, long-term DC intake has been shown to decrease PWV and increase flow mediated dilation (FMD).^26^^,^^27^ Acute DC intake has also been reported to increase FMD^28^; however, there are no reports of improved central arterial stiffness. In the study of Vlachopoulos et al,^16^ the group that consumed DC rich in cacao polyphenols, FMD increased significantly at 60 min after intake but cfPWV did not change. The main changes in arterial stiffness due to DC intake are suggested to occur in peripheral arterial vessels .^16^ DC intake increases NO bioavailability, which improves vascular smooth muscle tone,^13^ which may lead to improved vascular endothelial function and reduced arterial stiffness. It has been reported that epicatechin levels in the blood peak at 120 min after DC intake^15^^,^^29^ and that NO is also high 120 min after intake.^15^^,^^30^ Epicatechin also antagonizes NO and inhibits the production of the vasoconstrictor ET-1, which has been reported to decrease 120 min after DC intake.^30^^,^^31^ These mechanisms of action increase FMD 120 min after DC intake.^15^^,^^16^^,^^32^ As 30 min after RE corresponds to 120 min after DC intake, the present time course analysis suggests that the intake of DC had influenced the reduction of baPWV that had increased after the RE.

Peripheral arteries are composed predominantly of vascular smooth muscle cells whereas central arteries are mainly composed of elastic fibers. Acute DC intake has a greater effect on vascular smooth muscle cells than on elastic fibers, suggesting that the effects of acute DC intake are more likely to occur in the peripheral arteries. In fact, acute DC intake decreased peripheral arterial stiffness^28^ but did not alter central arterial stiffness.^16^ As the mechanism of action of DC intake on blood vessels is centered on vascular smooth muscle cells, the effect of acute DC intake may have contributed to the reduction in peripheral arterial stiffness, which is rich in vascular smooth muscle cells. Accordingly, DC intake before RE affected baPWV, which was increased by RE, but had no effect on arterial compliance or beta-stiffness index. While we interpreted the baPWV findings primarily in relation to peripheral arterial stiffness, it should be noted that baPWV reflects both central and peripheral arterial segments. Therefore, we cannot exclude the possibility that some of the observed changes may have included central arterial contributions. Further studies using region-specific PWV measures (e.g., carotid–femoral or carotid–radial PWV) are warranted to clarify the segmental vascular responses.

This study has several limitations. This study was categorized into two trials, DC intake trial and WC intake trial, but did not include a control group with no intake. This group should be added in future study. In addition, this study was conducted on healthy young adult males and may not be generalizable to women, obese persons, or the elderly. Moreover, we did not assess blood biomarkers (e.g., NO and/or endothelin-1 etc) which could have important effects on PWV change. Furthermore, the intervention was not blinded to either participants or researchers, which may have introduced potential bias. Nevertheless, the present findings provide the first evidence that intake of DC before RE may contribute to reductions in arterial stiffness that has been increased by RE. Further studies are needed to establish the role of DC in reducing arterial stiffness caused by RE. Additionally, since this study focused exclusively on upper-body resistance exercise, the results may not be generalizable to lower-body or whole-body resistance exercise protocols, which could elicit different vascular responses.

## 5 Conclusion

In conclusion, dark chocolate intake before high-intensity RE had the effect of rapidly reduced systemic arterial stiffness that had been increased by RE, but central arterial stiffness remained unchanged.

## Author contributions

Conceptualization: UH, TO.

Data curation: UH, YH.

Formal analysis: UH, YH.

Investigation: UH, YH, TO.

Methodology: UH, YH, MN, TO.

Project administration: UH, TO.

Supervision: TO.

Writing – original draft: UH, TO.

Writing – review & editing: MN, TO.

All authors read and approved the final version of the manuscript.

## Funding information

None.

## Conflicts of interest statement

The authors declare that they have no known competing financial interests or personal relationships that could have appeared to influence the work reported in this paper.
